# Safety and efficacy outcomes of the Xen45 Gel Stent use for refractory glaucoma: a surgery series from surgeon trainees at a tertiary teaching hospital

**DOI:** 10.1186/s40662-019-0171-0

**Published:** 2020-02-01

**Authors:** Karen Hong, John Lind, Arsham Sheybani

**Affiliations:** 1 Department of Ophthalmology and Visual Sciences, Washington University School of Medicine, St. Louis, MO USA; 2 Barnes-Jewish Center for Outpatient Health, 4901 Forest Park Avenue, St Louis, MO 63110 USA

**Keywords:** XEN, Investigational devices, Glaucoma, Gelatin stent, Ab interno surgery, Minimally invasive glaucoma surgery, Subconjunctival drainage, Gelatin microstent

## Abstract

**Background:**

To study the effect of an ab interno gelatin stent (XEN45 Gel Stent, Allergan Inc., Irvine, California, USA) on intraocular pressure (IOP) as placed by glaucoma fellowship trainees in eyes with refractory glaucoma.

**Methods:**

A prospective noncomparative study at a tertiary training center on 28 unique eyes undergoing ab interno gelatin stent implantation by glaucoma fellowship trainees. Data was collected at baseline and postoperatively at day 1, week 1, and months 1, 3, 5, and 12. Primary outcome was mean IOP change. Secondary outcomes included change in number of glaucoma medication classes and visual acuity. Safety outcomes included needling rates. Surgical success was defined by achieving ≥20% reduction in IOP with the same or fewer classes of antiglaucoma medications from baseline without the need for secondary surgical intervention and/or stent removal.

**Results:**

At baseline, 28.6% (8/28) of the subjects had prior failed incisional glaucoma surgery in a study population that was 54% African-American, with 78% with severe glaucoma (average mean deviation of − 14.58 dB). Thirteen subjects terminated their clinic visits before their 12-month postoperative visit, leaving 15 subjects for end point analysis. Average IOP went from 21.6 mmHg (range 12.0–31.0, SD 6.6) at baseline to 12.5 mmHg (range 7.0–19.0, SD 3.6), a 42.1% reduction (*p* < 0.007). All subjects decreased the number of medication classes they were taking with an average reduction of 3.8 (range 2–5, SD 0.9) to 1.3 (range 0–3, SD 1.0) classes, or a 65.8% decrease (*p* < 0.006). Crude surgical success was 80.0% for the 15 subjects that followed up at 12 months. The Kaplan-Meier cumulative probability of success for all 28 subjects at 12 months was 70.4% (95% CI: 44.7–85.8%). Regardless of the length of follow-up, 21.4% (6/28) met failure criteria: 3 subjects failed because they required secondary surgical intervention, and the other 3 did not have adequate IOP reduction. Initial bleb needling rate was 28.6% (8/28) and repeat was 17.9% (5/28).

**Conclusions:**

Compared to the reported literature with experienced ocular surgeons, ab interno gel stent placements by glaucoma fellowship trainees have similar mean IOP, topical medication reduction, surgical success, and needling rates at 12-month follow-up.

## Background

Glaucoma is the leading cause of irreversible blindness worldwide, currently affecting 64 million people globally and projected to impact 112 million by 2040 [1]. In primary open angle glaucoma (POAG), which accounts for approximately 70% of all glaucoma [1], the abnormal resistance is believed to be located along the outer aspect of the trabecular meshwork, which is the drainage system of the eye. The resistance to flow causes the intraocular pressure (IOP) to increase, which can result in damage to the optic nerve that can cause blindness. Therefore, the cornerstone of glaucoma treatment consists of IOP reducing medications, devices, and surgery, introduced in a step-wise fashion.

The treatment of glaucoma is approached in a step-wise fashion. Medication often is the initial treatment of choice aimed at lowering the IOP. If medication fails to adequately reduce the pressure or there are challenges to access and/or compliance, selective laser trabeculoplasty (SLT) may be performed. The drawback of SLT is that its effectiveness generally decreases over time [2].

If these conservative therapies fail, incisional surgeries such as trabeculectomies and aqueous tube shunts are considered. In a trabeculectomy, a flap is created over a hole that allows fluid inside the eye to drain beneath the conjunctiva. Aqueous shunt devices drain aqueous fluid through a silicone tube to the outer surface of the eye. These procedures are superior to medications at lowering IOP, but both are not known to be long lasting and can have devastating complications and failures. Reoperation may be needed for scarring that prevents the new filtration track from draining. One study showed a 40% failure rate 6 years after trabeculectomy [3], even with the administration of mitomycin C (MMC), an agent applied at the time of surgery to prevent scarring. Significant diplopia can occur if the aqueous shunt plate is placed too close to an ocular muscle. Due to the poor ability to control pressure fluctuations with these surgeries, both procedures carry a risk of blebitis, endophthalmitis, or choroidal hemorrhage.

Micro-invasive glaucoma surgery (MIGS) may have better safety profiles compared to traditional incisional glaucoma surgeries. The surgery minimizes conjunctival trauma and are usually placed under direct visualization of anterior chamber angle anatomy. Most MIGS use a clear corneal incision, known as an ab interno approach, to allow drainage of fluid through 1 of 4 pathways: 1) bypassing the trabecular meshwork, 2) increasing uveoscleral outflow through suprachoroidal pathways, 3) decreasing ciliary body aqueous or 4) creating a subconjunctival drainage pathway. Avoiding damage to the conjunctiva also allows the possibility of future glaucoma surgery. MIGS procedures are typically performed on patients with mild to moderate glaucoma because the amount of IOP reduction is not as great as traditional incisional approaches.

The XEN45 Gel Stent (Allergan Inc., Irvine, CA, USA) is a United States Food and Drug Administration (FDA)-approved device that drains excess fluid produced within the eye through the subconjunctival drainage pathway that bypasses trabecular and scleral resistance. Currently, it is the only device that mimics the non-physiologic drainage pathway of trabeculectomy and aqueous shunt surgeries while obviating the need for conjunctival dissection. The stent is comprised of a porcine gelatin crosslinked with glutaraldehyde that may be better accepted by human tissue and potentially minimize erosion. The gelatin also imparts hydrophilic properties that allow the implant to expand when hydrated by aqueous contact, which may help secure the device location after surgical implantation and prevent stent migration.

Given its relatively recent appearance on the market (FDA approval on November 21, 2016), the XEN45 MIGS procedure is not as commonly performed as incisional or other available MIGS procedures. The first prospective studies started with examination of the larger XEN63 and XEN140 devices [4, 5] and since then there have been prospective [6–11] and retrospective [12–15] studies examining the safety and efficacy of XEN45 Gel Stent placement. Overall, the consensus is that the XEN45 is effective in lowering IOP in patients with open angle glaucoma over the course of 12 months [16]. Complications are usually transient and mild to moderate in severity, with transient hypotony being the most commonly noted adverse event [4, 6–8]. Fortunately, complications are rare, although a suprachoroidal hemorrhage had been reported as a case study [17]. Stent failures required secondary surgical intervention and/or explantation [5–9]. All studies reported averages of > 20% reduction in IOP, and found needling to be a common intervention for bleb management [5–10].

There is a paucity of data on success rates of XEN45 Gel Stent implantations performed by surgical trainees. Marques et al. [18] published 1 study examining the first 6 gel stent implantations of 5 ophthalmology resident physician trainees who had performed over 250 cataract surgeries, compared to 5 experienced ophthalmic specialist surgeons with no prior XEN45 placement experience trained by Allergan (Allergan Inc., Irvine, California, USA). Both were found to have similar mean surgical times, but residents had more intraoperative and postoperative complications such as bleb hemorrhage, XEN45 placement and drainage issues, hypotony, and transitory inflammatory reactions. These complications decreased over the course of the residents’ first through sixth surgeries. The study did not compare pre- and postoperative IOP.

To the best of our knowledge, safety and efficacy studies of the XEN45 have not been characterized in ophthalmology fellowship trainees. As ab interno gelatin stent popularity increases, it will likely become a more common procedure practiced by residents and fellows at teaching institutions. Given the advantageous safety profile of MIGS compared to incisional surgeries, ophthalmic trainees should be familiar with a variety of MIGS procedures to provide the full spectrum of surgical options to their patients. The aim of this study is to describe the success rate, failures, and complications of the XEN45 stent placement as performed by glaucoma fellowship trainees at a tertiary teaching hospital. Those results will be compared to outcomes of experienced ophthalmic surgeons, as described in the literature.

## Methods

### Design

This prospective study was approved by the Institutional Review Board of the Washington University in St. Louis Human Research Protection Office and was conducted in accordance with tenets of the Declaration of Helsinki. All patients were provided written informed consent that was signed prior to enrollment into the study and received treatment in accordance with United States federal regulations. This study was compliant with the Health Insurance Portability and Accountability Act.

### Participants

Eligible subjects were identified from a pool of clinic patients 45 years and older who had refractory glaucoma and were recruited from January 1, 2016 to December 31, 2018. Refractory glaucoma was defined as previously failing a filtering or cilioablative procedure (e.g., cryotherapy, cyclodiode therapy), or subjects who had any other glaucomas that did not provide satisfactory results (including neovascular, congenital or infantile glaucomas) or IOP uncontrolled on maximum tolerated medical therapy (defined as a minimum of 4 or more classes of topical glaucoma medications or fewer if others were not tolerated or were ineffective). Anatomically, subjects were required to have an area of healthy, free and mobile conjunctiva in the target surgical quadrant, along with trabecular meshwork visible on gonioscopy, with Shaffer angle grade ≥ 3 in the target quadrant. Best-corrected visual acuity (BCVA) had to be light perception or better at their first preoperative visit. Subjects were required to have presence of a detectable glaucomatous field defect, defined as a mean deviation score of ≤ − 3 dB based on a SITA Standard 24–2 visual field analysis from a reliable, interpretable visual field exam (for eyes with BCVA better than 20/100). For BCVA of 20/100 or worse, no visual field eligibility criterion was required. Recruitment only occurred if the subject had the availability, willingness, and sufficient cognitive awareness to comply with examination procedures and the required visit schedule.Exclusion criteria were: angle closure glaucoma where the angle was not surgically opened, active neovascular glaucoma, clinically significant inflammation or infection, active uveitis, any corneal disease, central corneal thickness ≤ 490 μm or ≥ 620 μm, presence of vitreous in the anterior chamber, presence of intraocular silicone oil, active retinal disease, anticipated need for other ocular surgery in the 12 month follow-up period, fellow (non-study) eye with BCVA worse than 20/200, inability to discontinue contact lens wear, impaired episcleral venous drainage, history of dermatologic keloid formation, steroid use within the past 30 days that is not chronic, requiring anticoagulation therapy other than 81 mg aspirin a day at the time of surgery, recent chemotherapy, known or suspected allergy or sensitivity to porcine products or glutaraldehyde, fellow eye with an attempted non-study implantation procedure, concurrent participation in another drug or device clinical trial, and/or pregnant or nursing women.

### Surgical criteria

The XEN45 Gel Stent would not be implanted if any of the following were observed prior to implantation: 1) A lack of healthy conjunctiva showing free mobility (free of scarring or evidence of prior surgery) in the targeted quadrant. 2) Excessive intraoperative bleeding, such that visualization in the eye was impaired. 3) Any anatomy or finding in the eye that limited the investigator’s ability to visualize the anterior chamber, angle, or targeted quadrant of the conjunctiva. 4) Other surgical complication that in the opinion of the investigator could impede proper placement of the implant. Subjects who failed to meet the intraoperative eligibility criteria were converted to a different glaucoma intervention (e.g., trabeculectomy) prior to placement of the XEN45 Gel Stent and were exited from the study.

### Gelatin stent

The XEN45 Gel Stent is a microscopic hydrophilic tube composed of porcine dermis-derived gelatin, crosslinked to glutaraldehyde. Its dry inner and outer diameters are approximately 45 μm and 150 μm, respectively, with a length of 6 mm. The device comes preloaded in the sterile XEN Injector (Allergan Inc., Irvine, California, USA) for surgical placement of the stent. Details have been previously published on this device [19].

### Surgical technique

The XEN45 Gel Stent target quadrant area was injected with mitomycin C (MMC; Mitosol; Mobius Therapeutics LLC, St Louis, Missouri, USA). Needle cap and retention plug were removed from the top of the sterile preloaded XEN Injector containing the implant. Using an ab interno approach, the surgeon advanced the tip of the needle through the peripheral cornea, just anterior to the limbus, and across the anterior chamber to the target. Once the tip of the needle was aligned with the desired entry point into the trabecular meshwork, the surgeon advanced the needle through the trabecular meshwork and sclera. The surgeon was then able to directly visualize the entire beveled needle tip as it exits the sclera into the subconjunctival space with the surgical microscope. When the beveled tip of the needle reached the subconjunctival space, the surgeon released the implant by moving the slider forward. Once the implantation procedure was complete, the surgeon removed and discarded the injector. The participant took the following postoperative medications: fluoroquinolone (four times a day, on week 1 following surgery) and prednisolone acetate 1% (four times a day for weeks 1–4; three times a day on week 5; two times a day on week 6; every day for weeks 7–12). The glaucoma fellows were ophthalmic surgeons who had graduated from ophthalmology residency and were undergoing glaucoma sub-specialization under the mentorship of an accredited glaucoma specialist. Trainees performed these surgeries over the course of 1 year at Washington University in St. Louis School of Medicine, in collaboration with Barnes-Jewish Hospital (St. Louis, Missouri, USA).

### Assessments and outcomes

The preoperative visit included a routine complete ophthalmic history and examination with visual field testing. The severity of glaucoma was classified as low, moderate, or severe based on visual field testing and the glaucoma definition in the 10th revision of the International Statistical Classification of Diseases [20].

Postoperative visits occurred at day 1, weeks 1 (7 ± 2 days) and 2 (14 ± 3 days), and months 1 (28 ± 7 days), 3 (84 ± 14 days), 6 (182 ± 14 days), 8 (238 ± 14 days), 10 (294 ± 14 days), and 12 (375 ± 45 days). Postoperative visits included a routine history and examination and notation of any complications or needling procedures. IOP measurements were made by Goldmann applanation tonometer. Best corrected visual acuity and the number of anti-glaucomatous medications were recorded at all visits.

### Statistical analysis

The primary outcome was the mean IOP change from baseline to 12 months for all subjects who completed their baseline and 12-month follow-up exam.

Secondary outcomes included changes in mean IOP and mean number of antiglaucoma medications at 3, 6, and 12 months compared to baseline. Additional descriptors included BCVA, bleb needling rates, and success of XEN45 Gel Stent intervention. Success was defined as percentage of patients achieving ≥20% mean diurnal IOP reduction by 12 months on the same number or fewer antiglaucoma medications.

Safety outcomes included adverse events, glaucoma-related secondary surgical interventions, XEN45 explantations, and analysis of surgical failure.

One eye per subject was analyzed using STATA 14.2 (StataCorp LP, College Station, Texas, USA). If there were 2 surgical eyes from the same subject, only the first surgical eye was selected for analysis. Descriptive data were presented as means and standard deviations, or counts and percentages as appropriate. Wilcoxon signed-rank tests were used to compare means between quantitative variables with a *p* value of 0.05 or less considered statistically significant. For subjects with missing values, the subject’s last clinic visit was designated as the date of loss to follow-up. A Kaplan-Meier curve was plotted to express cumulative survival, with a failure defined as a glaucoma-related secondary surgical intervention with or without device explantation, device explantation alone, or not achieving ≥20% IOP reduction on the same number of medications or fewer by 12 months, similar to previous studies [7].

Primary and secondary outcomes were analyzed with subjects that had baseline and 12-month follow-up visits. Safety outcomes were analyzed with all included subjects. Subjects with missing data were censored at date of last follow-up for Kaplan-Meier survival analysis.

## Results

### Participants

There was a total of 28 patients (31 eyes) that were recruited in the study, with 3 eyes excluded because the first surgical eye was already included from the same patient. Preoperative baseline eye measurements and characteristics are found in Table 1. All subjects were either non-Hispanic White (46.0%) or African-American (54.0%). Most subjects had primary open angle (60.7%), juvenile open angle (14.3%), or normal-tension glaucoma (10.7%) with the most common prior glaucoma surgery being SLT. The majority of subjects had severe glaucoma (78.6%), defined as optic nerve findings consistent with glaucomatous visual field abnormalities in both hemifields and/or loss within 5 degrees of fixation in at least one hemifield [20]. Subjects had an average mean deviation on visual field testing of − 14.58 dB (SD 9.0 dB) at baseline preoperatively. There were 6 subjects (21.4%) that accounted for 8 prior failed incisional glaucoma surgeries (Table 1). One subject had a failed diode and 2 failed MIGS implantations (iStent and ExPRESS Mini Glaucoma Shunt with mitomycin C), all in one eye. All other subjects had one prior failed surgery each. There were no Gonioscopy-Assisted Transluminal Trabeculotomy or Dual Blade procedures performed on any of the eyes.

**Table 1:** Baseline Demographics and Characteristics

Demographic/Characteristic	*n* = 28 subjects
Mean Age, years (SD)	66.6 (11.0)
Female, n (%)	11 (39.3)
Ethnicity, n (%)	
Non-Hispanic White	13 (46.0)
Black/African American	15 (54.0)
Type of glaucoma, n (%)	
Primary Open Angle	17 (60.7)
Juvenile Open Angle	4 (14.3)
Normal Tension	3 (10.7)
Pseudoexfoliative	2 (7.1)
Uveitic	2 (7.1)
Preoperative lens status, n (%)	
phakic	20 (71.4)
pseudophakic	8 (28.6)
Total Prior Glaucoma Surgery, n (%)^a^	6 (21.4%)
Non-valved tube	2 (7.1)
ExPRESS shunt with MMC	2 (7.1)
Ahmed	1 (3.6)
TM iStent	1 (3.6)
Trabeculectomy	1 (3.6)
Diode	1 (3.6)
Goniotomy (GATT or KDB)	0 (0.0)
Total Prior Non-Glaucoma Ocular Procedures, n (%)	23 (82.1)
Laser Trabeculoplasty	21 (75.0)
Prior other non-phacoemulsification surgery ^b^	2 (7.1)
Glaucoma Severity^c^	
Severe	22 (78.6)
Moderate	2 (7.14)
Mild	4 (14.3)
Mean Deviation, dB (SD)	−14.58 (9.0)
Mean preoperative IOP, mmHg (range, SD)	21.7 (5.6)
IOP range mmHg	12–34
Mean Glaucoma Medication Classes^d^, n (SD)	3.7 (0.8)
1 class	0 (0.0)
2 classes	2 (7.1)
3 classes	7 (25.0)
4 classes	16 (57.1)
5 classes	3 (10.7)

*SD=* standard deviation; *MMC=* mitomycin C; *IOP=* intraocular pressure; *GATT=* gonioscopy-assisted transluminal trabeculotomy; *KDB=* Kahook Dual Blade

^a^One subject with 3 prior glaucoma surgeries: a non-valved tube, diode, and ExPRESS shunt with MMC

^b^IOL repositioning (*n* = 1), pars plana vitrectomy (*n* = 1)

^c^Glaucoma severity is described as optic nerve abnormalities consistent with glaucoma and the following categorization of glaucomatous visual field abnormalities: Severe = both hemifields and/or loss within 5 degrees of fixation in at least one hemifield, Moderate = glaucomatous visual field abnormalities in one hemifield and not within 5 degrees of fixation, Mild = no abnormalities or only present on short-wavelength automated perimetry or frequency doubling perimetry

^d^Oral carbonic anhydrase inhibitors are considered their own class

Most of the surgeries (82.1%) were completed by 1 of 4 fellows with the attending surgeon in the operating room, but 5 of the cases (17.9%) were completed by a glaucoma fellow on their own, independent of supervision. Table 2 lists other intraoperative variations.

**Table 2:** Intraoperative Characteristics^a^

Characteristic	*n* = 28 subjects
Operative eye (right eye), n (%)	14 (50.0)
Fellow-only procedure^b^	5 (17.9)
Combined phacoemulsification/XEN procedure, n (%)	9 (32.1)
MMC timing	
Before	27 (96.4)
After	1 (3.6)
MMC concentration	
0.2 mg/ml	27 (96.4)
0.4 mg/ml	1 (3.6)
Mean MMC total administration, mcg (SD)	37.9 (1.7)
20 mcg	3 (10.7)
30 mcg	4 (14.3)
40 mcg	19 (67.9)
60 mcg	2 (7.1)

^a^All procedures were completed using an ab interno approach with closed conjunctiva technique and injection of MMC. Air technique and Ophthalmic Viscosurgical Device dissection were not used in any of the cases

^b^Defined as a procedure performed by a glaucoma fellow without the supervision of an attending glaucoma specialist

*MMC=* mitomycin C; *SD=* standard deviation

Table 1, see end of document text file.

### Primary outcome

**Fig. 1:**
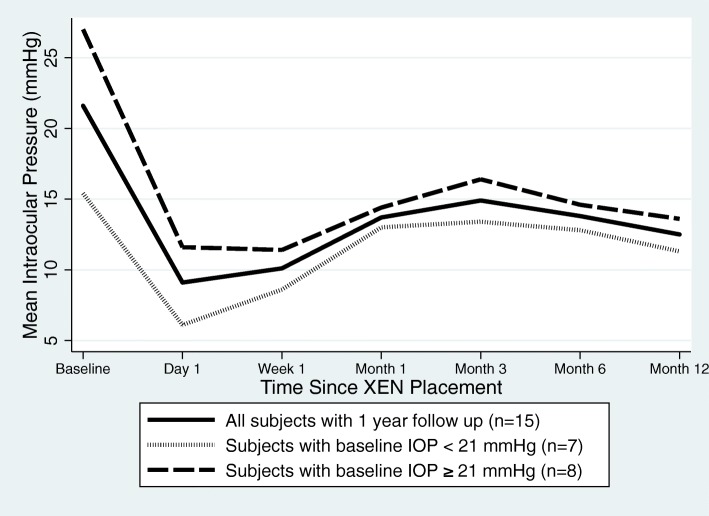
Intraocular pressure (IOP) at baseline and after XEN placement. The solid line is the mean of all subjects (*n* = 15). The dotted and dashed lines are subsets of the solid line, grouped by baseline IOP

All primary outcome data were calculated with IOPs recorded at baseline and the 12-month postoperative visit (*n* = 15 eyes). Preoperatively, patients had an average IOP of 21.6 mmHg (range 12.0–31.0, SD 6.6) that was reduced to 12.5 mmHg (range 7.0–19.0, SD 3.6) at 12 months postoperatively (*p* < 0.007). This was an average IOP reduction of 42.1% from preoperative baselines, or 9.1 mmHg (range 1.0–23.0, SD 6.1) (Fig. 1).

### Secondary outcomes

The primary outcome of average IOP change was also analyzed at 3 and 6 months, as those postoperative visits had more subjects completing the visits than at 12 months. At 3 months (*n* = 23) and 6 months (*n* = 19), there was an average IOP reduction of 21.7%, or 6 mmHg (range − 3.0–16.0, SD 5.2) and 35.0%, or 7.5 mmHg (− 3.0–20.0, SD 5.7), respectively.

**Fig. 2:**
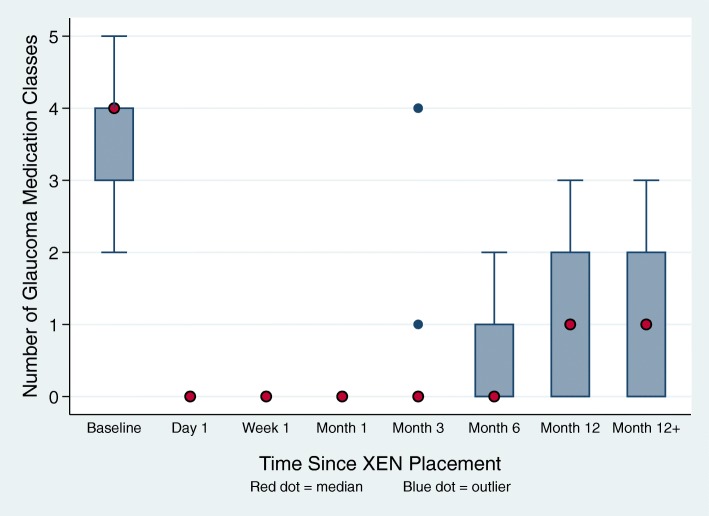
Number of glaucoma classes used by subjects at baseline and after XEN placement (*n* = 15). The interquartile range is from 25 to 75%, as indicated by the respective upper and lower borders of the boxes. The adjacent limits are 1.5 times the upper and lower limits of the respective interquartile ranges. Blue dots represent outliers. Red dots represent the median

There was a 65.8% decrease in the number of medication classes used 12 months after the intervention compared to the preoperative numbers at baseline (Fig. 2, *n* = 15). No medications were required for any of the patients up to 1 month postoperatively. Subjects were using an average of 3.8 (range 2–5, SD 0.9) medications classes prior to the operation and 1.3 classes (range 0–3, SD 1.0) 12 months later (*p* < 0.006). All subjects decreased the number of medication classes they were taking, and none increased at any point during the study.

The magnitude of reduction in the number of medication classes from baseline was even greater at 3 and 6 months compared to 12 months. At 3 months (*n* = 23) there was a 78.9% decrease in antiglaucoma medication classes while at 6 months (*n* = 19) there was a 76.3% decrease.

**Fig. 3:**
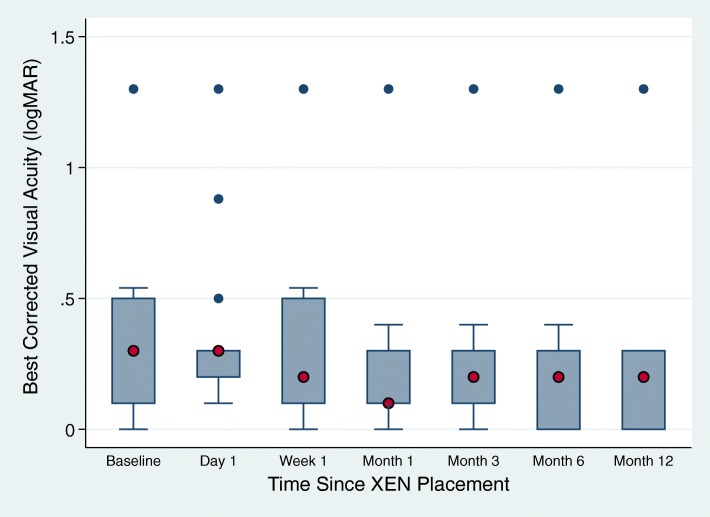
Best Corrected Visual Acuity at baseline and after XEN placement (*n* = 15). Blue dots represent outliers. Red dots represent the median. Interquartile range is from 25 to 75%, as indicated by the respective upper and lower borders of the boxes. The adjacent limits are 1.5 times the upper and lower limits of the respective interquartile ranges. Blue dots represent outliers. Red dots represent the median

The mean BCVA was slightly improved by one line 12 months postoperatively from an average starting Snellen chart vision of 20/40 to an ending average vision of 20/30 (*p* < 0.0034, Fig. 3). As expected, the day after surgery, there was an overall acute worsening of vision to an average of 20/60 with gradual improvement back to baseline or improvement from baseline visual acuity over the course of 12 months. Half (14/28) of the subjects had an improvement in visual acuity based on their last known clinical visit. More than a quarter (8/28) maintained their baseline vision and a minority (6/28) had worsened visual acuity. Of note, one subject who was 20/400 at baseline worsened to hand motion vision at 12 months.

The crude surgical success rate was 80.0% (12/15). All 3 subjects were considered surgical failures because they did not have adequate IOP reduction at 12 months. The subjects all initially had good IOP reductions after surgery, but one developed IOP < 20% reduction at 1 week, and the other 2 subjects failed at 1 month. However, all 3 subjects still had IOPs lower at 12 months when compared to their preoperative baseline IOP measurement.

**Fig. 4:**
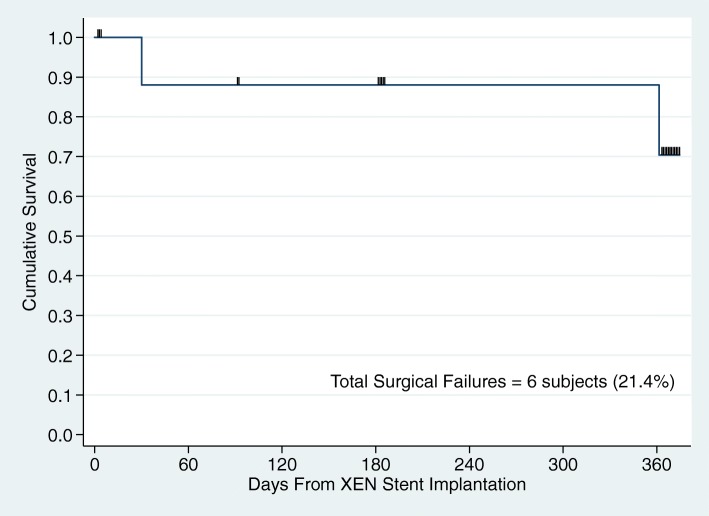
Kaplan-Meier survival curve (*n* = 28). There were initially 28 subjects at the beginning of the study. Of those, 13 were censored for loss to follow-up before 12 months. There were 6 subjects that were surgical failures

Upon accounting for censoring events, Fig. 4 shows the cumulative probability of surgical success was 70.4% (95% CI: 44.7–85.8%), as defined by achieving a 20% or more reduction in IOP with the same or fewer classes of antiglaucoma medications by 12 months, without the need for secondary surgical intervention and/or stent removal. There were 13 patients that were censored: 7 subjects for a last known clinic visit occurring before completing their 12-month postoperative visit and 6 subjects for returning to their referring provider once stable prior to 1 year of follow-up as they could no longer make the study visits.

### Surgical failures and adverse events

In total, there were 6 out of 28 subjects (21.4%) who failed their XEN45 implantation surgeries. There were 3 eyes (10.7%) that required secondary surgical intervention (Table 3). One of these failures was in an eye with moderate POAG with a malpositioned XEN Gel Stent 1 week postoperatively that developed into stent exposure 3 weeks later requiring device explantation and subsequent Molteno implantation. Another subject with severe pseudoexfoliation glaucoma required preoperative and postoperative needling procedures but continued to have a slowly increasing IOP to 40 mmHg, ultimately requiring an Ahmed valve placement. The third subject had severe juvenile open angle glaucoma with a prior ExPRESS shunt (Alcon Laboratories Inc., Fort Worth, TX) placement. Intraoperatively, this subject had a curled XEN45 as opposed to the usual straight appearance of the device. This curved implant may indicate that it is within tenon’s tissue instead of above the tenon’s which is our center’s desired implant location. The subject underwent a repeat XEN45 Gel Stent surgery after the first failed. The second XEN surgery was not included in the analysis. There were no surgical failures the day of surgery and there have been no other secondary surgical interventions or XEN explantations beyond one-year thus far.

**Table 3:** Surgical Outcomes Summary

	Subjects, n (%)
Total failure rate	6 (21.4)
Intraocular pressure not ≥20% reduction from baseline	3 (10.7)
Secondary surgical intervention	3 (10.7)
Postoperative Increase in antiglaucoma medications	0 (0.0)
Lost to follow-up prior to postoperative month 12^a^	13 (46.4)

^a^Patients lost to follow-up were censored in survival analysis

Table 4 describes the adverse events that occurred in the operating room and postoperative period. Trainees had a median of 9 months of glaucoma training at the time of implantation for the XEN45 devices that ultimately developed adverse events. Challenges intraoperatively were curled XEN Gel Stents (*n* = 3), a small orbit (*n* = 1), and a scarred superior conjunctiva (*n* = 1). These did not contribute to surgical failures other than the curled XEN45 described previously. Of note, there were 5 fellow-only cases total, and none of them had complications.

**Table 4:** Adverse events of XEN stent implant^a^

Adverse event^b^	Months in Fellowship Training at XEN placement	Initial Procedure
Intraoperative		
microhyphema	8	XEN
subconjunctival hemorrhage	5	XEN
Postoperative Day 1		
Anterior chamber tap for OVD removal	9	XEN-phacoemulsification
Descemet’s blockage at tube lumen	11	XEN-phacoemulsification
Postoperative Week 1		
Mild choroidal effusions	9	XEN
Serous choroidals	1	XEN
Low choroidals	9	XEN-phacoemulsification
Malpositioned XEN against iris	9	XEN
Malpositioned XEN pointing superiorly	11	XEN
Postoperative Month 1		
XEN Exposure	11	XEN

^a^All surgeries were performed with a supervising glaucoma attending

^b^All listed events were unique incidences that occurred one time. There were no noted adverse events on postoperative month 3, month 6, or month 12

*OVD=* ophthalmic viscoelastic device

### Postoperative interventions

There was 1 patient who required primary needling (day of surgery). The rate of postoperative bleb needling increased until 3 months postoperatively when the most needling and reneedling-procedures occurred (Table 5). The total rate of first-time needling was 28.6%. The rate of second time needling in the same patient was 17.9%.

**Table 5:** Interventional bleb needling rates over time

Time Postoperatively	First bleb needling n (%)	Median MMC concentration for initial needling (mcg)	Repeat Needling n (%)	Median MMC concentration for repeat needling (mcg)	Needling Complications (n)
Day of Surgery	1 (3.6)	60	0 (0.0)	–	Thick tissue (1)
Day 1	1 (3.6)	0	0 (0.0)	–	–
Week 1	1 (3.6)	0	0 (0.0)	–	–
Month 1	2 (7.1)	20	0 (0.0)	–	–
Month 3	2 (7.1)	10	3 (10.7)	20	Fractured XEN (1)
Month 6	1 (3.6)	5	1 (3.6)	20	Fractured XEN (1)
Month 12	0 (0.0)	0	0 (0.0)	–	–
Month 12+	0 (0.0)	0	1 (3.6)	0	–

*MMC=* mitomycin C

## Discussion

Traditional glaucoma surgery is currently not standardized and is invasive. The placement of the XEN45 Gel Stent involves a novel minimally invasive surgery allowing for a more standardized approach to lowering intraocular IOP because the device length and inner lumen are fixed. Its foremost advantage is placement without a large conjunctival incision like those required in traditional glaucoma surgeries. Here we report the device results when the surgery was performed by glaucoma fellowship trainees, which would inform the device’s potential in the hands of ophthalmic surgeons early in their surgical experience. Our results indicated a clinically significant 42.1% decrease in IOP from 21.6 mmHg to 12.5 mmHg, with a 65.8% reduction in medications in 15 eyes with follow-up at 1 year, suggesting that the XEN45 is a viable option for the surgical treatment of open angle glaucoma.

Subjects’ best corrected visual acuity remained at baseline on average. However, since these results are only over 12 months follow-up and correspond to 15 patients, this may be too short a period to observe vision changes.

Out of the 15 patients who had follow-up at 12 months, there was a crude 80.0% (12/15) surgical success rate which was higher than the cumulative probability found by Kaplan-Meier analysis (70.4%). This is likely because the survival curve examined all 28 subjects instead of only the 15 that had 12-month postoperative visits, allowing 3 additional surgical failures to be accounted for through censoring instead of being described as missing data like in the crude calculation.

There were 6 total surgical failures out of 28 subjects, 3 requiring secondary surgical intervention and the other 3 developing IOPs 20% over their baseline measurement. Adverse events included choroidal effusions (self-limited, serous, and low) and XEN malpositioning. These events centered around the first week and one-month postoperative visits. Needling procedures occurred the most during the third month postoperative visit. Overall, over a fourth of subjects required needling postoperatively and roughly 1 in 5 subjects required reneedling, making it common after XEN45 placement.

Despite the small sample size, additional analyses at 3 and 6 months where follow-up retention were consistent with the final 12-month follow-up findings. IOP reduction and glaucoma medication reduction were significant changes from the subjects’ preoperative baseline, indicating the feasible clinical application of the XEN45 device.

### Comparison to the literature

There have been several noncomparative prospective and retrospective studies [6–15] with consistent study-to-study trends even though the ranges for those effects varied broadly. In general, subjects’ IOPs started at a baseline in the low 20s and decreased to the low and mid-teens by the end of the first year from XEN45 Gel Stent placement. This was similar to our study’s finding.

The studies had an antiglaucoma medication class reduction range from 51.4 to 94.6% [4–10]. The trainees in our study had a 65.8% decrease in the number of classes for the 15 patients with 12-month follow-up, which fell within the range of what was previously reported. This suggests that outcomes are similar among trainees compared to reported outcomes of experienced surgeons.

Best corrected visual acuity outcomes were varied. Galal et al. [8] saw a two-line improvement with subjects, but part of this improvement may be due to the fact that over 30% of patients underwent the procedure in combination with cataract surgery. Other studies saw no statistically significant difference pre- and postoperatively [11], similar to our own findings.

Prior studies report a range of needling rates between 21.0–51.3% [4, 5, 7–9, 11, 12, 14, 15], with one reporting 2.4% [6]. Only 3 of the studies [4, 5, 15] mentioned that they used MMC or 5-fluorouracil during needling. This study’s postsurgical needling rate of 28.6% is within the range of the reported literature, but lower than most of the studies. The lower needling rate may be due to the amount of MMC administered at the time of surgery, but this is difficult to compare when the used concentrations vary per subject and study.

Despite the high preoperative glaucoma surgery failure rate of 21.4% among the participants, XEN45 placement as performed by trainees did not result in outcomes that differ greatly from experienced surgeons. Studies looking at surgical success rates ranged between approximately 60–80% [7–9, 12]. Our rate may have been on the higher end compared to the literature because the patients that did well likely continued returning to follow-up visits due to care satisfaction. Postoperative dosing of MMC may have also improved the overall success rate, as most prior studies either did not perform repeat dosing of MMC [8] or did not mention if it was administered concurrent with needling [6, 9, 11, 12, 14, 21]. Patients lost to follow-up were censored in the survival analysis since their outcome was unknown.

The results of our study conducted with glaucoma fellow trainees are consistent with the current reported literature of XEN45 Gel Stent outcomes as performed by experienced ophthalmic surgeons in terms of IOP, BCVA, and survival outcomes. It is notable that our outcomes were on a patient population with severe refractory glaucoma, suggesting that trainees can have good outcomes even when operating on advanced disease.

### Limitations

There were limitations to this study. First, this is a noncomparative study describing patient outcomes based on multiple different glaucoma fellows. A glaucoma specialist comparison group to the trainee glaucoma surgeons at the same institution may be able to elucidate subtle outcome changes between experienced and non-experienced surgeons.

Second, the large loss to follow-up of 13 patients in an already small sample size may over or underestimate our surgical success. The small sample size can cause large changes in estimates with each subject that was lost to follow-up. However, analysis at 3 months and 6 months had only 5 and 9 missing subject measurements, respectively, and both time points had decreased IOP and less antiglaucoma medication classes used, similar to the findings at 12 months. Of note, the percentage of IOP reduction increased from 3 to 6 to 12 months. Subjects with better outcomes may have been more inclined to follow-up for longer, leading to this result. Alternatively, the XEN45 may have become more effective over time, or the additional interventions of needling and reintroduction of antiglaucoma medications may have improved IOP control by 12 months. The reduction in antiglaucoma medications was greater at 3 and 6 months compared to 12 months, which could be due to the need for more antiglaucoma medications over time. Regardless, it is supportive that the reduction in need for antiglaucoma medications at 12 months is accurate, despite the small sample size. Even though the 3- and 6-month data were not primary end points, it supported the 12-month findings. Following outcomes beyond 12 months could elucidate whether subjects go back to their baseline medication class requirements over time.

Third, 7 of the subjects had concurrent phacoemulsification surgery, which makes it difficult to determine if their measured outcomes were due to the XEN45 Gel Stent, or removal of the cataract.

## Conclusion

The literature suggest that implantation of the XEN45 Gel Stent by an experienced surgeon is effective in lowering IOP in open angle glaucoma patients with infrequent adverse events that are almost always mild in severity and transient. Our results indicate that XEN45 Gel Stents can be implanted by a glaucoma trainee early in their surgical career with good success in reducing IOP by ≥20% at 12 months with similar outcomes and adverse events to experienced surgeons, even when treating severe glaucoma. Trainees should be proficient at bleb management postoperatively because needling interventions are commonly required after XEN45 Gel Stent placement.

The main concern with incorporating the XEN45 Gel Stent into the workflow of every glaucoma practice is that the long-term safety and efficacy of the device is still unknown. It is optimistic that early 12-month postoperative results demonstrate that the device is safe and effective in lowering both IOP and the number of antiglaucoma medications, with a high surgical success rate. Further studies looking at outcomes beyond 12 months would help determine the duration of these effects. Our results confirm the outcomes found in previous prospective noncomparative studies, and further suggest that the XEN45 Gel Stent implantation safety and efficacy features can be replicated even with young surgeon trainees.

## Data Availability

The datasets used and/or analyzed during the current study are available from the corresponding author on reasonable request.
